# Investigation of Solvolysis Kinetics of New Synthesized Fluocinolone Acetonide C-21 Esters—An *In Vitro* Model for Prodrug Activation

**DOI:** 10.3390/molecules16032658

**Published:** 2011-03-23

**Authors:** Bojan D. Markovic, Vladimir D. Dobricic, Sote M. Vladimirov, Olivera A. Cudina, Vladimir M. Savic, Katarina D. Karljikovic-Rajic

**Affiliations:** 1 Department of Pharmaceutical Chemistry, Faculty of Pharmacy, University of Belgrade, Vojvode Stepe 450, 11221 Belgrade, Serbia; 2 Department of Organic Chemistry, Faculty of Pharmacy, University of Belgrade, Vojvode Stepe 450, 11221 Belgrade, Serbia; 3 Department of Analytical Chemistry, Faculty of Pharmacy, University of Belgrade, Vojvode Stepe 450, 11221 Belgrade, Serbia

**Keywords:** prodrug, fluocinolone acetonide esters, solvolysis, descriptor, *in vitro* model

## Abstract

In this study the solvolysis of newly synthesized fluocinolone acetonide C-21 esters was analysed in comparison with fluocinonide during a 24-hour period of time. The solvolysis was performed in an ethanol-water (90:10 v/v) mixture using the excess of NaHCO_3_. The solvolytic mixtures of each investigated ester have been assayed by a RP-HPLC method using isocratic elution with methanol-water (75:25 v/v); flow rate 1 mL/min; detection at 238 nm; temperature 25 °C. Solvolytic rate constants were calculated from the obtained data. Geometry optimizations and charges calculations were carried out by Gaussian W03 software. A good correlation (R = 0.9924) was obtained between solvolytic rate constants and the polarity of the C-O2 bond of those esters. The established relation between solvolytic rate constant (K) and lipophilicity (cLogP) with experimental anti-inflammatory activity could be indicative for topical corticosteroid prodrug activation.

## 1. Introduction

Corticosteroids are used topically for their anti-inflammatory and immunosuppressive actions. Applied in this fashion, particularly to large areas, when the skin is injured, or under occlusive dressings, corticosteroids may be absorbed in sufficient amounts to cause systemic effects [1]. Therefore, the therapy with corticosteroids is limited both by systemic and local side effects. In order to increase benefit/risk ratio [2] the series of novel α-alkoxyalkanoyl and α-aryloxyalkanoyl esters with higher lipophilicity have been synthesized in our laboratory as analogues of fluocinolone acetonide 21-acetate (fluocinonide) [3]. Fluocinolone acetonide 21-acetate (FA-21-Ac) is about five times more potent than its active form fluocinolone acetonide (FA) due to enhanced lipophilicity and percutaneous absorption [4,5]. Thus, the new synthesized corticosteroid esters: fluocinolone acetonide 21-(2’-ethoxypropionate) (FA-21-EP), fluocinolone acetonide 21-(2’-ethoxybutyrate) (FA-21-EB), fluocinolone acetonide 21-(2’-methoxypropionate) (FA-21-MP), fluocinolone acetonide 21-(2’-methoxybutyrate) (FA-21-MB), fluocinolone acetonide 21-(2’-phenoxypropionate) (FA-21-PhP) could be potential excellent prodrug candidates to improve the drug’s physical and pharmaceutical properties. Prodrugs become pharmacologically active after the ester hydrolysis, since the glucocorticoid actions are caused only by corresponding glucocorticoid alcohols [6]. Thus, the hydrolysis of C17 or/and C21 esterified corticosteroids in biological media has a significant impact on their local potency and the systemic availability.

The hydrolysis of carboxylic acid esters is one of the most thoroughly studied chemical reactions in chemistry and biochemistry. Zhang *et al.* [7] have analyzed the hydrolysis of 41 different alkyl esters of aliphatic and aromatic carboxylic acids. They have calculated hydrolytic rate constants for all of the esters and established a model that correlates hydrolytic rate constant with several molecular descriptors (carbonyl carbon charge, carbonyl oxygen charge and LUMO energy). Methanol-mediated hydrolysis (e.g. methanolysis followed by hydrolysis) of *β*-lactams mimics the enzymatic pathway and could be studied in the gas phase and solution as a model reaction for hydrolysis of *β*-lactams by *β*-lactamases [8].

Our previous work was related to the application of derivative spectrophotometry for the study of FA-21-PhP solvolysis [9]. The aim of this study is to establish kinetic rate orders and the corresponding solvolytic rate constants of the novel esters and comparison with fluocinonide. Correlations between experimentally determined solvolytic rate constants and descriptors of steric and electronic properties of those esters have been established. The solvolytic model could be efficient as an *in vitro* model for corticosteroid C-21 ester prodrug activation. The significance of this model was established by obtaining a good correlation between the anti-inflammatory activity (determined in the test of inhibition of croton oil induced mice ear edema) [3] with solvolytic rate constants and lipophilicity. These correlations could be used in a rational design of novel esters.

## 2. Results and Discussion

### 2.1. Solvolytic rate constants determination

The solvolytic processes were followed after 0, 2, 4, 6, 8 and 24 hours using a HPLC method. Concentrations of fluocinolone acetonide (FA) in each solvolytic mixture were determined via FA peak areas, using the method of regression analysis. The concentration of corresponding ester in each solvolytic mixture was calculated using equation (1):

c(t) = c(0)−c
(1)

where: c(t) – concentration of corresponding ester after 2, 4, 6, 8 or 24 hours.
c(0) – concentration of corresponding ester in freshly prepared mixtures (t = 0)
c – concentration of FA (equal to the concentration of solvolyzed ester fraction)

**Figure 1:**
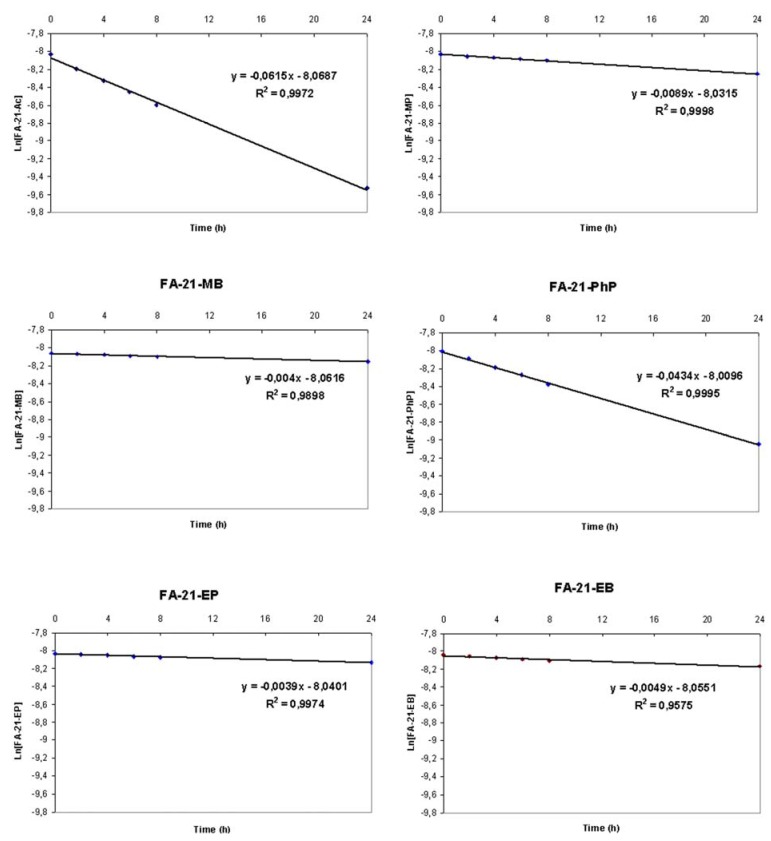
Solvolysis of six fluocinolone acetonide esters.

Solvolytic rate constants are obtained from the slope of the plot of ln[ester concentration] *versus* time, as shown in Figure 1.

### 2.2. Mechanism of solvolysis of fluocinolone acetonide esters

**Scheme 1:**
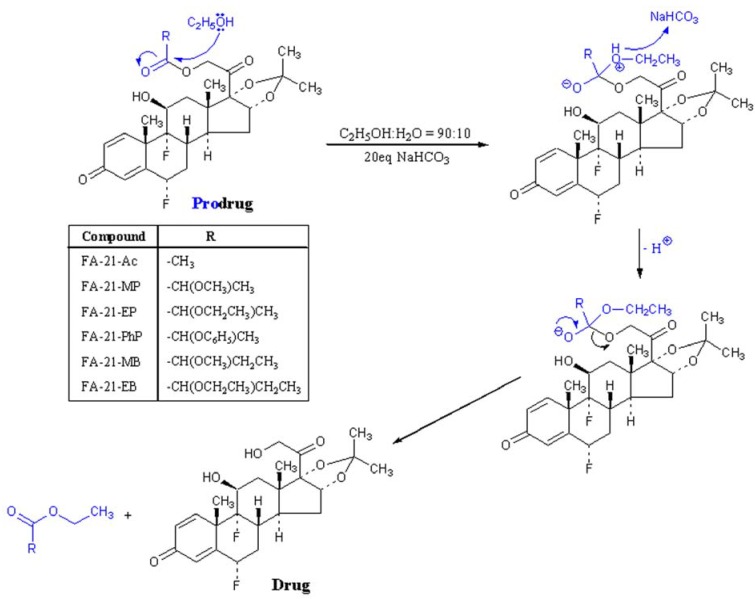
The general pathway of fluocinolone acetonide C-21 esters solvolysis.

Corticosteroid-21-esters are prodrugs and undergo *in vivo* hydrolysis by esterases. Products of THE hydrolysis are the pharmacologically active alcohol and the corresponding acid. Due to the poor water solubility of the analyzed FA esters, selected conditions of solvolysis included an ethanol-water (90:10 v/v) solvent mixture with an excess of sodium hydrogen carbonate. Under these conditions, the degradation products are the FA and the corresponding ethyl esters (transeesterification) [10,11,12]. The general pathway of fluocinolone acetonide C-21 ester solvolysis is shown in Scheme 1.

### 2.3. Kinetics of esters solvolysis

The graphs were plotted using ln[ester concentration] *versus* time (Figure 1) with denoted data of regression and correlation analysis. Correlation coefficients indicate good linearity. These results confirmed pseudo-first-order kinetics, which means that solvolytic rate depends on ester concentration when the concentration of NaHCO_3_ is significantly higher.

### 2.4. Descriptor selection

All the tested esters differ from each other in the acid used for esterification. Thus, their susceptibility to solvolysis can be affected by the steric, electronic or physico-chemical properties of corresponding acids. Considering this, following descriptors have been selected: cLogP, carbonyl carbon steric hindrance (steric hindrance), C=O1 bond length and C-O2 bond length, charge of carbonyl carbon (C), charge of ester oxygen (O2), charge of carbonyl oxygen (O1), charge differences C-O1, charge differences C-O2, polarity [13] of C-O2 bond (P_C-O2_), polarity of C=O1 bond (P_C=O1_). The values of calculated descriptors and their coefficients of correlation (R) with solvolytic rate constants (K) are presented in Table 1.

**Table 1:** Solvolytic rate constants (K) and descriptor values.

		FA-21-Ac	FA-21-EB	FA-21-EP	FA-21-MB	FA-21-MP	FA-21-PhP	**R**
****C-O2 bond length****		1.381	1.369	1.370	1.369	1.370	1.374	****0.9627****
****C=O1 bond length****		1.229	1.232	1.232	1.232	1.232	1.228	****-0.9168****
****AM1****	****C****	0.2991	0.2990	0.3062	0.2991	0.3069	0.2777	****-0.5215****
	****O2****	-0.2580	-0.2237	-0.2284	-0.2234	-0.2282	-0.2851	****-0.8342****
	****O1****	-0.2830	-0.2950	-0.2949	-0.2943	-0.2940	-0.3227	****-0.0956****
	****C-O2****	0.5571	0.5226	0.5346	0.5225	0.5351	0.5628	****0.9023****
	****C-O1****	0.5821	0.5940	0.6011	0.5934	0.6009	0.6004	****-0.5807****
	****P_C-O2_****	0.7694	0.7155	0.7324	0.7153	0.7331	0.7732	****0.9247****
	****P_C=O1_****	0.7154	0.7318	0.7405	0.7311	0.7403	0.7373	****-0.6644****
****HF/STO-3G gas phase****	****C****	0.3080	0.2971	0.3027	0.2975	0.3033	0.3025	****0.7510****
	****O2****	-0.2595	-0.2475	-0.2515	-0.2471	-0.2511	-0.2767	****-0.7309****
	****O1****	-0.2407	-0.2489	-0.2478	-0.2481	-0.2468	-0.2575	****0.1229****
	****C-O2****	0.5676	0.5446	0.5542	0.5446	0.5544	0.5792	****0.8315****
	****C-O1****	0.5488	0.5460	0.5505	0.5457	0.5502	0.5599	****0.4620****
	****P_C-O2_****	0.7838	0.7455	0.7592	0.7456	0.7595	0.7958	****0.8771****
	****P_C=O1_****	0.6745	0.6727	0.6782	0.6723	0.6778	0.6876	****0.3561****
****HF/6-31G gas phase****	****C****	0.7654	0.7898	0.8195	0.7917	0.8212	0.8161	****-0.4782****
	****O2****	-0.6927	-0.6792	-0.6837	-0.6784	-0.6833	-0.7509	****-0.5802****
	****O1****	-0.5207	-0.5390	-0.5423	-0.5383	-0.5413	-0.5598	****0.2239****
	****C-O2****	1.4582	1.4689	1.5033	1.4701	1.5044	1.5670	****0.1414****
	****C-O1****	1.2862	1.3288	1.3618	1.3300	1.3625	1.3759	****-0.4076****
	****P_C-O2_****	2.0137	2.0110	2.0595	2.0126	2.0611	2.1531	****0.2631****
	****P_C=O1_****	1.5807	1.6371	1.6778	1.6386	1.6786	1.6896	****-0.4605****
****B3LYP/STO-3G gas phase****	****C****	0.2314	0.2233	0.2277	0.2237	0.2283	0.2232	****0.4277****
	****O2****	-0.1879	-0.1783	-0.1807	-0.1778	-0.1802	-0.2010	****-0.7492****
	****O1****	-0.2095	-0.2172	-0.2167	-0.2165	-0.2157	-0.2207	****0.4194****
	****C-O2****	0.4193	0.4016	0.4084	0.4015	0.4085	0.4242	****0.8752****
	****C-O1****	0.4409	0.4405	0.4443	0.4402	0.4440	0.4439	****-0.0451****
	****P_C-O2_****	0.5790	0.5498	0.5595	0.5496	0.5596	0.5828	****0.9118****
	****P_C=O1_****	0.5418	0.5427	0.5474	0.5423	0.5470	0.5452	****-0.3450****
****B3LYP/6-31G gas phase****	****C****	0.5089	0.5138	0.5387	0.5167	0.5410	0.5518	****-0.0727****
	****O2****	-0.4682	-0.4582	-0.4610	-0.4574	-0.4605	-0.5214	****-0.5609****
	****O1****	-0.3784	-0.3959	-0.3997	-0.5167	-0.3988	-0.4059	****0.4213****
	****C-O2****	0.9771	0.9720	0.9997	0.9742	1.0016	1.0731	****0.3315****
	****C-O1****	0.8874	0.9097	0.9383	1.0334	0.9398	0.9577	****-0.4469****
	****P_C-O2_****	1.3494	1.3306	1.3696	1.3336	1.3722	1.4745	****0.4106****
	****P_C=O1_****	1.0906	1.1207	1.1560	1.2732	1.1578	1.1760	****-0.4686****
****B3LYP/6-31G gas phase****	****C****	0.5089	0.5138	0.5387	0.5167	0.5410	0.5518	****-0.0727****
	****O2****	-0.4682	-0.4582	-0.4610	-0.4574	-0.4605	-0.5214	****-0.5609****
	****O1****	-0.3784	-0.3959	-0.3997	-0.5167	-0.3988	-0.4059	****0.4213****
	****C-O2****	0.9771	0.9720	0.9997	0.9742	1.0016	1.0731	****0.3315****
	****C-O1****	0.8874	0.9097	0.9383	1.0334	0.9398	0.9577	****-0.4469****
	****P_C-O2_****	1.3494	1.3306	1.3696	1.3336	1.3722	1.4745	****0.4106****
	****P_C=O1_****	1.0906	1.1207	1.1560	1.2732	1.1578	1.1760	****-0.4686****
****HF/6-31G CPCM ethanol****	****C****	0.3188	0.3057	0.3086	0.3062	0.3089	0.3107	****0.9101****
	****O2****	-0.2633	-0.2597	-0.2616	-0.2591	-0.2612	-0.2736	****-0.6169****
	****O1****	-0.2719	-0.2765	-0.2765	-0.2752	-0.2748	-0.2745	****0.8871****
	****C-O2****	0.5821	0.5655	0.5702	0.5654	0.5701	0.5843	****0.9243****
	****C-O1****	0.5907	0.5822	0.5850	0.5815	0.5837	0.5852	****0.8641****
	****P_C-O2_****	0.8038	0.7741	0.7811	0.7740	0.7810	0.8028	****0.9578****
	****P_C=O1_****	0.7260	0.7173	0.7208	0.7164	0.7192	0.7186	****0.7311****
****B3LYP/STO-3G COSMO ethanol****	****C****	0.2370	0.2277	0.2287	0.2280	0.2293	0.2281	****0.7636****
	****O2****	-0.1910	-0.1877	-0.1883	-0.1872	-0.1881	-0.1979	****-0.6912****
	****O1****	-0.2395	-0.2424	-0.2426	-0.2943	-0.2940	-0.3227	****-0.0345****
	****C-O2****	0.4280	0.4154	0.4171	0.4153	0.4174	0.4260	****0.9853****
	****C-O1****	0.4766	0.4701	0.4713	0.5223	0.5233	0.5508	****0.1160****
	****P_C-O2_****	0.5911	0.5687	0.5714	0.5685	0.5719	0.5853	****0.9924****
	****P_C=O1_****	0.5857	0.5791	0.5807	0.6435	0.6447	0.6764	****0.0966****
****Steric hindrance****		1.24	1.36	1.34	1.37	1.35	1.40	****-0.5356****
****cLogP****		2.79	3.79	3.26	3.40	2.87	4.59	****0.0533****
****K****		0.0615	0.0049	0.0039	0.0040	0.0089	0.0434	****1.0000****

### 2.6. Relationships between solvolytic rate constant and selected descriptors

There are two mechanisms for base-catalyzed hydrolysis: B_AC_2 (base-catalyzed, acyl oxygen cleavage, bimolecular) and B_AL_2 (base-catalyzed alkyl oxygen cleavage, bimolecular). In most cases, B_AC_2 prevails over the B_AL_2 reaction path [14,15]. In accordance with the fact that solvolysis is a similar process to hydrolysis and with respect to the B_AC_2 mechanism, the carbonyl carbon is the site of the ethoxide ion attack and the cleavage site for alcoholic group formation, any changes at the carbonyl group will directly affect the rate of solvolysis. In the following study the C(=O1)-O2 moiety and its properties were used to correlate reaction rate data. Since many substituents affect charge distribution of carbonyl group, they can have impact on solvolysis rate. Therefore, different acids used for corticosteroid esterification, can affect ester stability and solvolysis rate. 

Starting from AM1 optimized structures the best correlations were obtained between solvolytic rate constant and C-O2 distance (R = 0.9627). Slight changes in bond length could affect bond dissociation. A similar correlation (R = 0.9247) has been found between solvolytic rate constants and polarity of C-O2 bonds. In order to find the most accurate model *ab initio* Hartree-Fock (HF) and Density Functional Theory (DFT) with B3LYP model calculations have been performed. In these calculations different basis sets (STO-3G and 6-31G) were used. To assess the influence of solvatation effects these calculations have been carried out in the gas phase and in the solvent - ethanol (the conductor-like screening model COSMO and the conductor-like polarizable continuum model CPCM). 

Analyzing the data from Table 1 a certain correlation between solvolytic rate constant and carbonyl carbon charge could be noted. If the carbonyl carbon charge is higher, nucleophilic ethoxide ion will attack it easily. Since this phase of base-catalyzed ester solvolysis is rate limiting, higher carbonyl charge accelerates this process (R = 0.9101 in HF/6-31G computation model using CPCM solvatation method with ethanol as solvent). Among the tested FA esters, FA-21-Ac and FA-21-PhP have significantly higher solvolytic rate constants and corresponding carbonyl carbon charges than the other esters. Acetic acid has three hydrogen atoms in the α-position which negligibly affect its carbonyl carbon charge, whereas α-phenoxypropionic acid has a group with positive (methyl) and strong negative inductive effect (phenoxy) and the overall effect is electron withdrawing, which increases carbonyl carbon charge. In the molecules of acids which have been selected for synthesis (α-methoxybutyric, α-methoxypropionic, α-ethoxypropionic and α-ethoxybutyric acid) there is one group in the α-position with a negative inductive effect (methoxy or ethoxy group) and one group with positive inductive effect (methyl or ethyl group). These groups have opposite effects on carbonyl carbon charge and make it less positive comparing to carbonyl carbon of acetic and α−phenoxypropionic acids.

**Figure 2:**
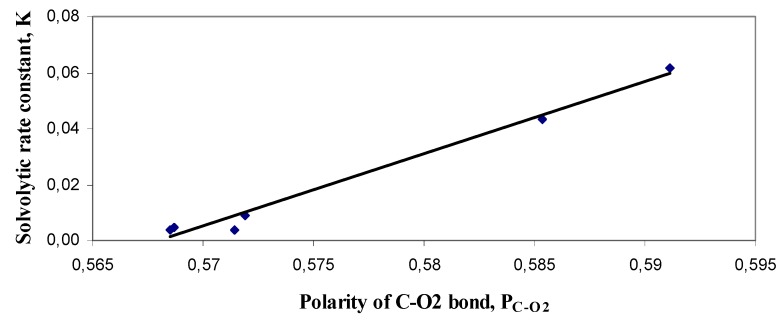
Correlation between polarity of C-O2 bond (B3LYP/STO-3G SCRF(COSMO, ethanol)) and solvolytic rate constant.

Gas phase *Ab initio* calculations with different levels of theory and different basis sets did not give significantly better correlations than semi-empirical AM1. However, good correlations were achieved if these calculations are carried out in the presence of solvent. Correlation analysis showed that the most accurate model was obtained by using polarity of C-O2 bond calculated by B3LYP/STO-3G model with ethanol as a solvent in COSMO solvatation model (Figure 2). The polarity presents a descriptor calculated by multiplication of charge differences with the distance between two atoms and a good correlation polarity *versus* rate of hydrolysis of *N*-acetylazoles has been obtained [13]. In our study, polarity of C-O2 band (P_C-O2_) was calculated by multiplication of charge differences between carbonyl carbon charge (C) and ester oxygen charge (O2) with C-O2 bond length (bond length measured on AM1 optimized structures, Mulliken charges calculated by B3LYP/STO-3G model using ethanol as solvent in COSMO solvatation model).

The equation (2) was obtained using single linear regression analysis:

K = (2.5808±0.1604) × P_C-O2_ – (1.4658 ± 0.0924) (2)

where R = 0.9924, SE = 0.0035, F = 258.855; K – pseudo-first order solvolytic rate constant; P_C-O2_ – polarity of C-O2 bond 

### 2.7. Relationship between solvolitic rate constant and anti-inflammatory activity

**Figure 3:**
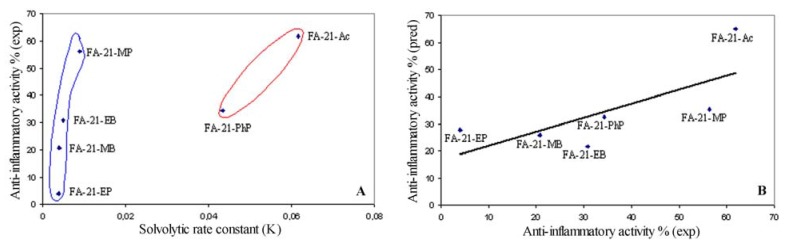
A. Experimental anti-inflammatory activity *versus* solvolytic rate constant. B. Predicted *versus* experimental anti-inflammatory activity of fluocinolone acetonide C-21 esters.

Solvolysis kinetics of the new synthesized fluocinolone acetonide C-21 esters have been investigated as a simple, rapid and low cost *in vitro* model for topical corticosteroid prodrug activation. Anti-inflammatory activity (represented as a percentage of reduction of mass edema) has been determined in a test of croton oil induced mice ear edema. To assess whether this model can be used to estimate the prodrugs activation, a regression analysis between local anti-inflammatory activity and solvolytic rate constants was performed. There is no significant correlation between these two parameters, but it could be noted that there are two groups (Figure 3A) – the first with high solvolysis rate constants (FA-21-Ac and FA-21-PhP) and the second with low solvolysis rate constants (FA-21-MP, FA-21-EP, FA-21-MB and FA-21-EB). Topical prodrug activity depends on several factors: rate of hydrolysis, interactions of the active form with target structures, permeability, *etc.* James *et al*. [16] studied the effect of various aliphatic esters of testosterone on rat prostate and seminal vesicles and correlated with lipophilicity and rate of ester hydrolysis by liver esterase. In accordance with this manuscript anti-inflammatory activity was linked (Figure 3B) with solvolysis rate constant (K) and lipophilicity (cLogP – values are of significance ranged from 2.79 to 4.59).

Good correlation (R = 0.7214) obtained by multiple regression analysis is indicative that this *in vitro* solvolytic model could be used as a model for topical corticosteroid prodrugs activation. 

## 3. Experimental

### 3.1. General

Fluocinolone acetonide 21-acetate (FA-21-Ac) was purchased from Galenika a.d. Serbia. Fluocinolone acetonide 21-(2’-ethoxypropionate) (FA-21-EP), fluocinolone acetonide 21-(2’-ethoxy-butyrate) (FA-21-EB), fluocinolone acetonide 21-(2’-methoxypropionate) (FA-21-MP), fluocinolone acetonide 21-(2’-methoxybutyrate) (FA-21-MB) and fluocinolone acetonide 21-(2’-phenoxy-propionate) (FA-21-PhP) were synthesized in our laboratory. Synthesis of the fluocinolone acetonide esters was carried out using racemic α-alkoxyalkanoyl or α-aryloxyalkanoyl acids thus affording the corresponding mixtures of two diastereomeric products. Acids used for esterifications, as well as the corresponding esters, are in the form of racemic mixtures. The diastereomers did not show separated signals in ^1^H-NMR spectra, most likely due to their overlap. Lack of mutual interactions between the acid and the steroid moieties caused by the distance between them is presumably the reason for the observed ^1^H-NMR results. On the other hand, ^13^C-NMR spectra clearly suggested the presence of two diastereomeric products, although it is difficult to differentiate them unambiguously. 

Reagents: fluocinolone acetonide, absolute ethanol Chromasolv HPLC purity, methanol Chromasolv HPLC purity (Sigma-Aldrich Chemie GmbH), sodium hydrogen carbonate, thionylchloride (Merck), ethyl 2-bromopropionate, 2-bromobutiric acid, 2-phenoxypropionic acid (Fluka), dichlormethane, ethyl acetate (Carlo Erba), N,N-dimethylformamide (Alfa Aesar) and deionized water (TKA water purification system, Niederelbert, Germany) were used throughout this study.

Melting points were determined on a Boetius PHMK 05 apparatus. ^1^H-NMR and ^13^C-NMR spectra were recorded on a Bruker AVANCE instrument at 500 and 125 MHz using CDCl_3_ as a solvent and TMS as an internal standard, chemical shifts (δ) are given in ppm, coupling constants (*J*) in Hz. IR spectra were recorded on a Thermo Scientific NICOLET iS10 by using diamond ATR. Direct ESI-MS spectra were recorded on Agilent Technologies 6210-1210 TOF-LC-ESI-MS instrument in positive mode. 

### 3.2. General procedure for synthesis of fluocinolone acetonide α-alkoxyalkanoyl and α-aryloxy-alkanoyl C-21 monoesters

In this study five structurally related acids were used for the esterification of FA. 2-Methoxypropionic, 2-ethoxypropionic, 2-methoxybutyric and 2-ethoxybutyric acid were prepared from the corresponding 2-bromopropionic and 2-bromobutyric acids with sodium methoxide or sodium ethoxide, according to the patent literature [17]. The mentioned acids were transformed into the corresponding chlorides by reaction with an excess of thionyl chloride. Due to high volatility of prepared acylchlorides, the losses resulting compounds were significant during removal of the last traces of thionyl chloride, so the yields of pure compounds were about 15%. Typically, to a solution of FA (220.0 mg, 0.486 mmol) in dichloromethane (15 mL) triethylamine (0.1 mL, 0.721 mmol) was added. The mixture was cooled at 0 °C and corresponding acyl chloride (134.6 mg, 0.729 mmol) was added dropwise. The resulting mixture was stirred 4 h at room temperature. Dichloromethane (50 mL) was added and the solution washed with hydrochloric acid (1 M, 50 mL), aqueous solution of sodium hydrogen carbonate (5%, 50 mL) and water (twice with 50 mL) and dried with anhydrous Na_2_SO_4_. The crude product was purified by silica gel chromatography using chloroform as mobile phase to afford the pure product.

*(6α,11β,16α)-6,9-Difluoro-11,21-dihydroxy-16,17-[(1-methylethylidene) bis(oxy)]-pregna-1,4-diene-3,20-dione-21-(2'-ethoxypropionate)* (**FA-21-EP**). White solid, melting point 231–232.5 °C. ^1^H-NMR: 0.94 (3H, d, *J* = 7 Hz, H-18), 1.23 (3H, s, H-23), 1.44 (3H, s, H-24), 1.47 (3H, dd, *J* = 17 Hz, *J* = 7 Hz, H-2’’), 1.53 (3H, s, H-19), 1.59-2.01 (7H, m, H-7α, H-12β, H-15, H-3’), 2.15–2.56 (4H, m, H-7β, H-8, H-12α, H-14), 3.49–3.72 (2H, m, H-1’’), 4.09–4.15 (1H, m, H-2’), 4.42 (1H, d, *J* = 8 Hz, H-11), 4.90–5.03 (3H, m, H-16, H-21), 5.32–5.46 (1H, dq, *J* = 48.5 Hz, *J* = 6 Hz, H-6), 6.37 (1H, dd, *J* = 10.5 Hz, *J* = 1.5 Hz, H-2), 6.44 (1H, s, H-4), 7.12 (1H, d, *J* = 10 Hz, H-1). ^13^C-NMR: 15.2, 16.3, 18.9, 23.0, 23.1, 25.8, 26.5, 29.7, 31.8, 31.9, 32.0, 33.6, 33.8, 34.0, 37.1, 42.7, 45.6, 47.9, 48.0, 65.8, 67.8, 67.9, 71.6, 71.9, 74.5, 81.8, 85.6, 87.1, 97.4, 97.9, 99.3, 111.8, 121.2, 121.3, 130.3, 150.3, 161.1, 173.5, 185.4, 203.2. IR (ATR) 857.3, 896.7, 934.3, 996.2, 1,068.5, 1,128.3, 1,160.7, 1,209.9, 1,265.6, 1,374.1, 1,455.3, 1,609.1, 1,627.5, 1,666.8, 1,727.9, 2,925.1, 3,333.8 cm^-1^. MS [M+H]^+^ calculated for C_29_H_39_F_2_O_8_ = 553.26075; observed = 553.26104.

*(6α,11β,16α)-6,9-Difluoro-11,21-dihydroxy-16,17-[(1-methylethylidene) bis(oxy)]-pregna-1,4-diene-3,20-dione-21-(2'-ethoxybutyrate)* (**FA-21-EB**). White solid, melting point 255–257 °C. ^1^H-NMR: 0.94 (3H, t, H-18), 1.00–1.06 (2H, m, H-4’) 1.23–1.27 (3H, s, H-23), 1.44 (3H, s, H-24), 1.53 (3H, s, H-19), 1.59–2.01 (6H, m, H-7α, H-12β, H-15, H-3’), 2.13–2.56 (4H, m, H-7β, H-8, H-12α, H-14), 3.49–4.20 (2H, m, H-1’’), 3.90–4.21 (1H, m, H-2’), 4.42 (1H, t, *J* = 3 Hz, H-11), 4.93–5.01 (3H, m, H-16, H-21), 5.32–5.46 (1H, dq, *J* = 48.5 Hz, *J* = 6 Hz, H-6), 6.37 (1H, dd, *J* = 10 Hz, *J* = 2 Hz, H-2), 6.43 (1H, s, H-4), 7.12 (1H, d, *J* = 10 Hz, H-1). ^13^C-NMR: 9.6, 15.1, 16.3, 18.14, 23.0, 25.7, 26.3, 26.4, 26.5, 26.9, 31.7, 31.8, 31.9, 32.0, 33.4, 33.6, 33.8, 34.0, 37.1, 37.4, 42.7, 45.3, 45.6, 48.0, 59.7, 63.8, 66.1, 67.4, 67.8, 71.5, 71.8, 79.8, 81.6, 81.8, 85.6, 87.1, 97.3, 98.0, 99.4, 111.7, 121.2, 121.5, 130.2, 150.4, 161.2, 172.9, 185.5, 203.2. IR (ATR) 856.6, 897.6, 934.9, 996.4, 1,068.0, 1,179.9, 1,264.0, 1,374.5, 1,626.7, 1,664.9, 1,725.9, 2,937.8, 3,336.1 cm^-1^. MS [M+H]^+^ calculated for C_33_H_39_F_2_O_8_ = 567.27640; observed = 567.27683.

*(6α,11β,16α)-6,9-Difluoro-11,21-dihydroxy-16,17-[(1-methylethylidene)bis(oxy)]-pregna-1,4-diene-3,20-dione-21-(2'-methoxypropionate)* (**FA-21-MP**). White solid, melting point 236–238 °C. ^1^H-NMR: 0.94 (3H, d, J=10, H-18), 1.22 (3H, s, H-23), 1.44 (3H, d, *J* = 2.5 Hz, H-24), 1.53 (3H, s, H-19), 1.60–2.22 (7H, m, H-7α, H-12β, H-15, H-3’), 2.28–2.55 (4H, m, H-7β, H-8, H-12α, H-14), 3.45 (3H, d, *J* = 3.5 Hz, H-1’’), 4.03 (1H, m, H-2’), 4.42 (1H, m, H-11), 4.85–5.01 (3H, m, H-16, H-21), 5.32–5.46 (1H, dq, *J* = 49 Hz, *J* = 6.5 Hz, H-6), 6.37 (1H, 1H, dd, *J* = 10 Hz, *J* = 1.5 Hz, H-2), 6.44 (1H, s, H-4), 7.12 (1H, dd, *J* = 10.5 Hz, *J* = 1.5 Hz, H-1). ^13^C-NMR: 16.3, 18.5, 20.5, 23.0, 23.1, 25.8, 26.5, 29.7, 31.7. 31.8, 31.9, 32.0, 33.6, 33.8, 34.0, 37.1, 42.7, 45.5, 45.6, 47.9, 48.1, 57.8, 67.6, 71.6, 71.9, 76.2, 81.8, 85.6, 87.1, 97.4, 98.0, 99.4, 111.7, 111.8, 121.2, 121.3, 130.2, 150.4, 161.1, 161.2, 170.7, 185.5, 203.6. IR (ATR) 859.5, 897.6, 994.0, 1,038.7, 1,068.2, 1,130.2, 1,161.1, 1,181.5, 1,209.3, 1,265.4, 1,233.7, 1,374.6, 1,408.1, 1,455.5, 1,610.3, 1,627.4, 1,667.4, 1,727.2, 1,746.3, 2,940.0, 3350.8 cm^-1^. MS [M+H]^+^ calculated for C_33_H_39_F_2_O_8_ = 539.24450; observed = 539.24450.

*(6α,11β,16α)-6,9-Difluoro-11,21-dihydroxy-16,17-[(1-methylethylidene) bis(oxy)]-pregna-1,4-diene-3,20-dione-21-(2'-methoxybutyrate)* (**FA-21-MB**). White solid, melting point 224–225 °C. ^1^H-NMR: 0.95 (3H, d, *J* = 7 Hz, H-18), 1.02 (3H, dt, *J* = 15 Hz, *J* = 5 Hz, H-4’), 1.23 (3H, s, H-23), 1.44 (3H, s, H-24), 1.54 (3H, s, H-19), 1.6–1.97 (6H, m, H-7α, H-12β, H-15, H-3’), 2.17–2.56 (4H, m, H-7β, H-8, H-12α, H-14), 3.45 (3H, d, *J* = 3 Hz, H-1’’), 3.83-3.88 (1H, m, H-2’), 4.43 (1H, t, *J* = 3 Hz, H-11), 4.91–5.07 (3H, m, H-16, H-21), 5.32–5.46 (1H, dq, *J* = 50 Hz, *J* = 4 Hz, H-6), 6.37 (1H, dd, *J* = 10, *J* = 1.5 Hz, H-2), 6.44 (1H, s, H-4), 7.14 (1H, d, *J* = 10 Hz, H-1). ^13^C-NMR: 9.3, 16.3, 23.0, 25.8, 26.1, 26.5, 30.9, 31.8, 32.0, 33.6, 34.0, 37.0, 42.7, 45.6, 47.9, 48.1, 58.1, 67.8, 71.5, 71.8, 81.4, 81.8, 85.6, 87.1, 97.4, 98.0, 99.4, 111.7, 121.1, 121.2, 130.2, 150.6, 161.2, 161.3, 172.6, 185.5, 203.2. IR (ATR) 857.3, 896.5, 996.3, 1,067.2, 1,131.2, 1,262.8, 1,243.6, 1,374.4, 1,456.1, 1,627.1, 1,665.0, 1,727.3, 2,938.4, 3,336.4 cm^-1^. MS [M+H]^+^ calculated for C_33_H_39_F_2_O_8_ = 553.26075; observed = 553.26135. 

*(6α,11β,16α)-6,9-Difluoro-11,21-dihydroxy-16,17-[(1-methylethylidene) bis(oxy)]-pregna-1,4-diene-3,20-dione-21-(2'-phenoxypropionate)* (**FA-21-PhP**). White solid, melting point 222–224 °C. ^1^H-NMR: 0.89 (3H, d, *J* = 24.5 Hz, H-18), 1.22 (3H, s, H-23), 1.43 (3H, s, 24H), 1.50 (3H, d, *J* = 4 Hz, H-19), 1.59–1.80 (7H, m, H-7α, H-12β, H15, H-3’), 1.99 (1H, bs, OH), 2.13–2.50 (4H, m, H-7β, H-8, H-12α, H-14), 4.37 (1H, bs, H-11), 4.88 (1H, q, *J* = 3 Hz, H-2’), 4.93–5.02 (3H, m, H-16, H-21), 5.37 (1H, qd, *J* = 48.7 Hz, *J* = 3 Hz, H-6), 6.35 (1H, d, *J* = 10 Hz, H-2), 6.42 (1H, s, H-4), 6.92 (2H, m, H-2’’, H-6’’), 6.99 (1H, m, H-4’’), 7.09 (1H, dd, *J* = 10 Hz, *J* = 1.5 Hz, H-1), 7.29 (2H, t, *J* = 8 Hz, H-3’’, H-5’’). ^13^C-NMR: 16.3, 18.7, 23.0, 25.8, 26.5, 29.7, 31.8, 33.6, 33.8, 34.0, 37.03, 42.7, 45.6, 47.9, 68.0, 71.6, 72.1, 81.8, 85.6, 87.1, 97.3, 97.9, 99.3, 111.8, 115.0, 115.1, 121.2, 121.7, 129.6, 130.2, 150.4, 161.2, 172.2, 185.5, 203.0, IR (ATR) 693.3, 858.9, 900.6, 936.3, 969.1, 999.9, 1,067.5, 1,132.8, 1,186.8, 1,232.8, 1,297.5, 1,373.5, 1,385.8, 1,447.3, 1,496.0, 1,603.4, 1,665.3, 1,729.0, 1,765.9, 2,946.0, 3,302.0 cm^-1^. MS [M+H]^+^ calculated for C_33_H_39_F_2_O_8_ = 601.26075; observed = 601.25983.

### 3.3. Solvolytic process evaluation method

HPLC analysis were performed on a HP 1100 (Agilent Technologies, Palo Alto, CA, USA) system equipped with binary pump, Rheodyne injector (sample loop 20 μL), 1100 UV detector, controlled by IBM PC Pentium Vectra XA computer. Analyses were made on Zorbax Extend-C18 column (150 mm × 4.6 mm, i.d., 3.5 μm) Agilent, USA, using isocratic elution with methanol-water (75:25 v/v) at a flow rate of 1 mL/min. The experiments were conducted at 25 °C. The data were collected and processed using the Chem Station software package. Solvolysis sample mixtures were stirred with a Tehtnica Rotamix SHP-10 (Zelezniki, Slovenia) magnetic stirrer.

Stock aqueous solution of sodium hydrogen carbonate was prepared by dissolving NaHCO_3_ (280 mg) in a 50 mL volumetric flask (0.067 M).

*Preparation of FA stock solution (for calibration curve)* – accurately weighted FA (8 mg) was quantitatively transferred into a 5 mL volumetric flask with ethanol (approximate volume 3 mL) for complete dissolution and the flask was filled up to mark with ethanol (solution A). Aliquots of 0.1, 0.4, 0.7, 1 and 1.3 mL of solution A were transferred separately into five 10 mL volumetric flasks followed by ethanol (6 mL). NaHCO_3_ stock aqueous solution (1 mL) was added and each flask was filled up to mark with ethanol.

*Preparation of solvolysis sample mixtures* – accurately weighted FA-21-Ac (16 mg), FA-21-MB (18.5 mg), FA-21-MP (17.7 mg), FA-21-EB (18.35 mg), FA-21-EP (18 mg) and FA-21-PhP (20 mg) were quantitatively transferred into separate 100 mL volumetric flasks with ethanol (approximately volume 60 mL) for complete dissolution, NaHCO_3_ stock aqueous solution (10 mL) was added and the flasks were filled up to mark with ethanol. The prepared mixtures were immediately vigorously stirred (200 rpm).

For HPLC analysis aliquots of each solvolysis sample mixture (20 μL) were withdrawn and injected at time intervals 0, 2, 4, 6, 8 and 24 h. Chromatograms were recorded immediately using the UV detector set at 238 nm. The solvolysis percentages were calculated via the obtained peak areas corresponding to FA contents in the solvolytic mixtures after 0, 2, 4, 6, 8 and 24 h.

### 3.3. Computational detail

Initially, molecular mechanic optimizations of corticosteroid C-21 esters’ structures using MM2 algorithm have been carried out using Chem3D Ultra 7.0.0 software (Cambridge, MA, USA). The MM2 optimized structures were used for further optimization by semi-empirical AM1 method [18] implemented in Gaussian W03 [19]. AM1 optimized structures was used for calculation lengths of C=O1 and C-O2 bonds. Mulliken charges on carbonyl carbon (C) and both oxygen atoms of ester moiety (O1 and O2) were calculated using AM1 method. In order to find the most accurate model the *ab initio* Hartree-Fock (HF) [20] and Density Functional Theory (DFT) calculations with the hybrid exchange functional of Becke [21,22] and the Lee, Yang, and Parr [23] correlation functional (B3LYP) have been performed. In these calculation different basis sets (STO-3G and 6-31G) were used. To assess the influence of solvatation effects these calculations have been carried out in the gas phase and in the solvent - ethanol (the conductor-like screening model COSMO and the conductor-like polarizable continuum model CPCM). Steric hindrance is calculated in MarvinSketch 5.1.3 (ChemAxon Ltd., Budapest, Hungary). Lipophilicity (cLogP) has been calculated using ChemDraw Ultra 7.0.0 software (Cambridge, MA, USA).

### 3.4. Determination of anti-inflammatory activity in the test of inhibition of croton oil induced mice ear edema

Topical anti-inflamatory activities of fluocinolone acetonide C-21 esters have been determined by measuring inhibition of croton oil induced mice ear edema. Animals have been divided in groups of five mice. Croton oil solution in acetone (35 μg/mL) has been applied topically to the inner aspect of the right ear in a dose of 20 μL using a Hamilton syringe. Acetone (20 μL) has been applied in the left ear. Solutions of test compounds in acetone (28 μM) have been applied only to the right ear in doses of 20 μL, 30 minutes after croton oil application. The control group only received the solution of croton oil in the right ear, but no solution of corticosteroid ester. Four hours later, animals were sacrificed with diethyl ether and 6 mm diameters disks were cut from the middle part of ears. Anti-inflammatory activities were expressed as percentages of mass reduction of edema. The results have been recently published [3]. 

## 4. Conclusions

Corticosteroid esters, such as fluocinolone acetonide 21-esters, are excellent prodrug candidates to improve the parent drug’s physical and pharmaceutical properties. The solvolysis of new synthesized FA-21-esters was analyzed using rapid, simple and sensitive HPLC method. Solvolytic rate constants are found to be in good correlation with polarity of C-O2 bond (P_C-O2_) indicating that choice of acid used for prodrug synthesis affects ester stability and pharmacological action. This study shows that this *in vitro* solvolytic model obtained by multiple regression analysis between solvolytic rate constant and lipophilicity with anti-inflammatory activity, could be used as a model for prediction of prodrug activation.
